# Prevalence and correlates of hazardous, harmful or dependent alcohol use and drug use amongst persons 15 years and older in South Africa: Results of a national survey in 2017

**DOI:** 10.4102/phcfm.v13i1.2847

**Published:** 2021-03-23

**Authors:** Supa Pengpid, Karl Peltzer, Shandir Ramlagan

**Affiliations:** 1 ASEAN Institute for Health Development, Mahidol University, Salaya, Phutthamonthon, Nakhon Pathom, Thailand; 2 Department of Research Administration and Development, University of Limpopo, Polokwane, South Africa; 3 Department of Human and Social Capabilities, Human Sciences Research Council, Pretoria, South Africa

**Keywords:** alcohol use, drug use, adolescents, adults, health variables, South Africa

## Abstract

**Background:**

Harmful alcohol and illicit drug use significantly contribute the burden of disease.

**Aim:**

This study aimed to assess the prevalence and correlates of hazardous, harmful or dependent alcohol (HHDA) use and drug use amongst persons 15 years and older in South Africa.

**Setting:**

Population-based survey.

**Method:**

In a national cross-sectional 2017 survey, 39 210 persons 15 years and older (Median = 34 years) responded to a questionnaire on substance and health variables. The prevalence of HHDA use was 10.3% and past 3-month drug use 8.6%.

**Results:**

In adjusted logistic regression analysis, men of middle age (25–34 year olds) with higher education, urban residence, drug use and psychological distress were positively associated and Indian or Asian and white population groups were negatively associated with HHDA. Women of middle age (25–34 year old) and mixed race, residing on rural farms and urban areas, with drug use and psychological distress were positively associated and older age (55 years and older) and Indians or Asians were negatively associated with HHDA. In adjusted logistic regression analysis, men, having Grade 8–11 education, mixed race, being unemployed, and the HHDA used were positively associated and middle and older age (25 years and older) and being a student or learner were negatively associated with past 3-month any drug use. Women, who were mixed race, Indians or Asians, with the HHDA use were positively associated and older age (45 years and older) were negatively associated with the past 3-month drug use.

**Conclusion:**

About one in 10 participants with several sociodemographic and health indicators was identified to be associated with HHDA and any drug use.

## Introduction

Harmful alcohol and illicit drug use is a significant contributor to the global burden of disease.^1,2,3^ Globally, alcohol use contributed to 5.3% of all deaths and 5.0% of all disability-adjusted life years (DALYs) in 2016.^1^ The corresponding figures for South Africa in 2000 were 7.1% and 7.0%, respectively.^4^ Diverse alcohol use patterns have been observed in African countries.^5,6,7,8^ In a 2015 national survey in Kenya, 6.7% of the adult population engaged in hazardous or harmful alcohol use.^9^ In a 2008 national population-based survey of persons 15 years and older in South Africa, the prevalence of hazardous, harmful or dependent alcohol (HHDA) use was 9.0%, 17.0% amongst men and 2.9% amongst women.^10^

The estimated global past-year prevalence of illicit drug use was 5.3% in 2014^2^ and 3.8% for cannabis, 0.77% for amphetamines, 0.37% for opioid and 0.35% for cocaine use in 2015.^11^ In the 25 country World Mental Health Survey, ‘lifetime drug use disorders prevalence increased with country income: 0.9% in low-/lower-middle income countries, 2.5% in upper-middle income countries and 4.8% in high-income countries’.^12^ In Nigeria, the past-year prevalence of illicit drug use amongst adults was 14.4%.^13^ In a 2012 national population-based survey in persons 15 years and older in South Africa, the prevalence of past 3-month drug use was 4.4% (4.0% for cannabis use, 0.4% sedatives, 0.3% opiates, 0.3% amphetamines, 0.2% inhalants and 0.1% hallucinogens use in the past 3 months).^14^ There is a lack of more recent national population-based data on the prevalence and correlates of HHDA and drug use in South Africa.

As previously reviewed,^14,15^ factors associated with HHDA and/or drug use may include male sex, middle adulthood, specific ethnic groups, lower socioeconomic status, unemployed, urban residence and other substance use. In addition, several studies have shown the comorbidity of HHDA with drug use and psychological distress,^16,17^ as well as the comorbidity of drug use with HHDA and psychological distress.^6^ Epidemiological population-based surveys are needed to target interventions to prevent HHDA and drug use. The study aimed to assess the prevalence and correlates of HHDA and drug use amongst persons 15 years and older in South Africa.

## Methods

### Study design and participants

The data utilised in this study were obtained from a cross-sectional, nationally representative household-based survey conducted in 2017 in South Africa. The multistage stratified random cluster sampling approach of the survey is described elsewhere.^18^ In summary, the mid-year population estimates^19^ were utilised to select 1000 small area layers (SALs) that were stratified by province, locality type and race groups. A maximum of 15 households were randomly selected from each of the 1000 SALs. In each household, all household members, who resided in that household the previous night, were eligible to participate.^18^

### Study procedure

All eligible household members had to individually complete an informed consent form in private with the study fieldworker prior to being enrolled into the study. All questions that the respondent had during consent or interview were answered by the fieldworker or team supervisor. The respondent had the option to end the interview at any time without consequence. The household head or delegated household authority completed a household questionnaire, which captured demographic and household situation information and each individual in the household completed an individual questionnaire.^18^ The survey questionnaire was captured electronically by the fieldworker on a Mercer A105 tablet utilising Census and Survey Processing System (CSPro) software. Data were collected from December 2016 to February 2018. For this paper, data from the household and individual questionnaires were used. We restricted the sample to those who were 15 years and older and who completed the alcohol use measurement.

### Measures

#### Substance use variables

Hazardous, harmful or dependent alcohol was assessed using the Alcohol Use Disorders Identification Test (AUDIT)^20^ and was scored as in a previous survey in South Africa.^10^ Amongst adults (20 years and above), the cut-off score is 8 or more^20^ and amongst adolescents (15–19 years) 5 or more^21^ for classifying HHDA use. Cronbach’s alpha for the AUDIT was 0.87 in this sample.

*Drug use* in the past 3 months was assessed with seven items of the ‘Alcohol, Smoking and Substance Involvement Screening Test (ASSIST)’, for example, ‘In the past 3 months, how often have you used cannabis (dagga, marijuana, pot, grass, hash, etc.?’.^22^ One item was added ‘Whoonga (mixture of heroin, dagga = cannabis and antiretrovirals)’ and classified under opiates.^14^ ‘Response options ranged from 1 = never to 5 = almost daily. Any drug used in the past 3 months was coded as 1 and never as 0’.^14^ ‘All items were added together to indicate the prevalence of any drug use in the past 3 months’.^14^ Cronbach alpha for the ASSIST in this sample was 0.91.

*Sociodemographic factors* included age, sex, highest educational level, population group (African black, Coloured, Indian or Asian, white and others), employment status, province and residence status.^18^ Statistics South Africa asks people to describe themselves in the census in terms of five racial population groups, which is useful because of relevant differences between these population groups in terms of various health and other indicators.^10^

*Psychological distress* was assessed with the Kessler Psychological Distress Scale (K10), with scores 20 or more, indicating psychological distress.^23^ Cronbach’s alpha for the K10 was 0.92 in this sample.

### Ethical considerations

Approval for the survey was granted by the ‘Human Sciences Research Council (HSRC) Research Ethics Committee (REC: 4/18/11/15)’. Approval was also granted by the Centers for Disease Control and Prevention (CDC’s) Center for Global Health (CGH). Written informed consent was obtained from all participants.

### Data analysis

All statistical analyses were conducted using Statistics and data (STATA) software version 14.0 (Stata Corporation, College Station, TX, United States). The data were weighted to make the sample representative of the target population in South Africa. Descriptive statistics were used to summarise the sample and substance use prevalence characteristics. Unadjusted and adjusted (including variables significant at *p* < 0.05 in univariate analysis) logistic regression stratified by sex was used to predict HHDA and past 3-month drug use prevalence. Taylor linearisation methods were applied to account for the complex study design and the sampling weight. Results from logistic regression analyses are reported as odds ratios (ORs) and 95% confidence intervals (CIs). Missing values were excluded and *p* < 0.05 was considered significant.

## Results

### Characteristics of the sample and substance use

The sample comprised 39 210 persons 15 years and older (Median = 34 years, interquartile range = 25–48): 48.3% were men and 51.7% were women, 36.1% had Grade 12 or more education education and 79.3% were African black by population group or ethnicity. More than one in three participants (36.0%) were employed or self-employed, 69.0% lived in urban areas and 20.4% reported psychological distress. More than one in 10 respondents (10.3%) engaged in HHDA, 16.5% amongst males and 4.6% amongst females, and past 3-month drug use was 8.6%, 13.3% amongst males and 4.1% amongst females (see Table 1).

**TABLE 1:** Sample characteristics and distribution of substance use.

Variable	Sample		Hazardous, harmful or dependent alcohol use			Any drug use		
			Total	Male	Female	Total	Male	Female
		
	*N*	%	%	%	%	%	%	%
All	39 210	-	10.3	16.5	4.6	8.6	13.3	4.1
**Sex**								
Female	23 112	51.7	-	-	-	-	-	-
Male	16 098	48.3	-	-	-	-	-	-
**Age in years**								
15–17	3852	7.1	4.4	6.1	2.7	6.3	8.4	4.0
15–24	10 863	24.1	9.5	13.5	5.5	10.2	15.4	5.1
25–34	8749	27.2	14.9	23.0	6.8	11.1	17.9	4.5
35–44	6523	19.2	10.4	16.6	4.1	7.9	12.0	3.7
45–54	5315	13.1	9.0	14.7	3.8	6.4	9.6	3.5
55 or more	7760	16.5	5.1	10.2	1.6	4.4	6.2	3.1
**Education**								
Grade 0–7	18 901	37.3	7.8	11.8	4.2	7.0	10.5	4.0
Grade 8–11	9871	26.5	13.2	20.8	5.6	11.3	19.1	3.7
Grade 12 or more	12 362	36.1	11.0	17.9	4.3	8.1	12.0	4.4
**Population group**								
African black people	30 675	79.3	10.4	17.2	4.2	8.4	13.7	3.6
mixed race	4303	8.8	13.5	19.4	10.9	10.8	17.2	6.4
Indian or Asian people	2310	2.9	5.5	5.7	1.1	8.9	8.9	4.5
White people	1922	8.9	7.4	11.2	3.3	7.4	8.6	5.9
**Employment status**								
Employed/self-employed	11 931	36.0	12.2	17.4	4.8	8.9	12.3	4.1
Unemployed	20 649	50.2	9.7	17.5	4.5	8.8	15.9	4.0
Student/pupil/learner	5400	12.4	8.0	10.9	5.1	6.7	8.9	4.6
Sick/disabled/unable/others	748	1.4	8.7	14.6	2.6	6.7	9.7	3.7
**Residence**								
Rural informal	13 675	26.0	6.2	11.6	1.9	7.1	11.7	3.6
Rural farms	4263	5.0	9.6	12.3	5.8	8.8	12.2	4.2
Urban	21 372	69.0	12.0	18.5	5.7	9.1	14.0	4.3
**Province**								
Western Cape	2860	12.2	14.4	19.7	9.2	9.9	14.9	5.1
Eastern Cape	2970	10.7	8.0	12.6	4.0	4.8	8.3	1.7
Northern Cape	2030	2.0	15.3	22.7	7.9	11.3	16.5	6.0
Free State	1753	5.1	15.2	23.6	7.2	10.3	17.0	4.1
KwaZulu-Natal	13 512	18.6	4.5	7.8	1.9	8.9	14.9	4.0
North-West	2498	6.8	13.0	22.3	4.5	8.3	11.3	5.6
Gauteng	6183	27.2	13.0	20.3	5.3	9.3	14.4	4.0
Mpumalanga	5054	7.9	7.8	11.7	3.7	7.0	8.7	5.4
Limpopo	2350	9.5	8.3	15.3	2.3	8.3	13.9	3.5
**Psychological distress**								
No	31 307	79.6	9.8	15.6	4.1	8.3	12.8	3.8
Yes	7750	20.4	12.2	20.6	6.3	9.5	16.0	5.0

### Distribution of past 3-month drug use pattern

The most common drug used was cannabis (7.8%), 12.4% amongst males and 3.5% amongst females. The prevalence of cocaine use was 1.8%, followed by sedeatives 1.7%, amphetamine 1.5%, inhalents 1.3%, hallucinogens 1.2% and opiates 1.2% (see Table 2).

**TABLE 2:** Demographic distribution of the prevalence of past 3 month drug use.

Variable	Cannabis	Cocaine	Amphetamine	Inhalants	Sedatives	Hallucinogens	Opiates
	%	%	%	%	%	%	%
All	7.8	1.8	1.5	1.3	1.7	1.2	1.2
**Sex**							
Female	3.5	1.5	1.4	1.4	1.7	1.3	1.5
Male	12.4	2.1	1.5	1.2	1.7	1.2	2.0
**Age in years**							
15–17	6.0	1.4	1.2	1.2	1.4	1.2	1.8
15–24	9.5	2.1	1.8	1.6	1.7	1.6	2.2
25–34	10.0	1.9	1.6	1.3	2.0	1.2	1.7
35–44	7.0	1.6	1.2	1.1	1.5	1.0	1.6
45–54	5.8	1.5	1.3	1.4	1.8	1.3	1.6
55 or more	4.1	1.3	1.1	1.1	1.5	1.0	1.2
**Population group**							
African black people	7.8	1.8	1.5	1.4	1.7	1.3	1.9
mixed race	10.3	1.1	1.6	0.7	1.9	0.4	0.7
Indian or Asian people	6.1	2.5	1.5	1.4	2.1	1.6	1.7
White people	6.3	1.5	1.2	1.1	1.8	1.2	1.0
**Residence**							
Rural informal	6.6	2.2	1.7	1.6	1.6	1.5	1.9
Rural farms	7.7	1.4	1.2	1.4	1.6	1.2	1.6
Urban	8.3	1.6	1.4	1.2	1.8	1.2	1.6
**Province**							
Western Cape	8.9	1.5	1.4	0.6	1.6	0.7	1.1
Eastern Cape	4.4	0.6	0.7	0.4	0.6	0.4	0.9
Northern Cape	10.8	2.4	1.9	1.6	2.3	1.6	2.4
Free State	9.6	1.1	0.9	1.0	1.9	1.0	1.2
KwaZulu-Natal	8.2	2.4	2.1	2.0	2.2	1.9	2.2
North-West	7.3	2.6	2.7	2.8	3.1	2.5	3.4
Gauteng	8.5	0.9	0.6	0.6	1.1	0.5	1.0
Mpumalanga	6.4	3.3	3.0	3.2	2.9	2.9	3.3
Limpopo	7.4	3.0	1.6	1.6	1.7	1.4	2.1

### Associations with hazardous, harmful or dependent alcohol

In adjusted logistic regression analysis, amongst men who are middle age (25–34 year olds) with higher education, urban residence, drug use and psychological distress were positively associated, whereas Indian or Asian and white population groups were negatively associated with HHDA. Amongst women who are middle age (25–34 year olds), mixed race and residing on rural farms and urban areas with drug use and psychological distress were positively associated and older age (55 years and older) and Indians or Asians were negatively associated with HHDA (see Tables 3 and 4).

**TABLE 3:** Associations with hazardous, harmful or dependent alcohol consumption amongst men.

Variable	Simple logistic regression			Multiple logistic regression		
	Crude OR	95% CI	*p*	Adjusted OR	95% CI	*p*
**Age in years**						
15–24	1	Reference	-	1	Reference	-
25–34	2.33	1.91, 2.85	< 0.001	1.82	1.42, 2.32	< 0.001
35–44	1.55	1.22, 1.97	< 0.001	1.29	0.96, 1.74	0.092
45–54	1.34	1.03, 1.75	0.030	1.23	0.87, 1.74	0.233
55 or more	0.88	0.68, 1.15	0.345	0.89	0.65, 1.22	0.468
**Education**						
Grade 0–7	1	Reference	-	1	Reference	-
Grade 8–11	2.18	1.75, 2.71	< 0.001	1.52	1.18, 1.97	< 0.001
Grade 12 or more	1.79	1.44, 2.33	< 0.001	1.40	1.05, 1.87	0.024
**Population group**						
African black people	1	Reference	-	1	Reference	-
mixed race	1.04	0.83, 1.30	0.747	0.97	0.77, 1.51	0.823
Indian or Asian people	0.50	0.37, 0.69	< 0.000	0.40	0.28, 0.57	< 0.001
White people	0.62	0.46, 0.83	< 0.001	0.52	0.35, 0.78	< 0.001
**Employment status**						
Employed/self-employed	1	Reference	-	1	Reference	-
Unemployed	1.00	0.83, 1.19	0.960	1.14	0.92, 1.42	0.230
Student/pupil/learner	0.41	0.30, 0.54	< 0.001	1.93	0.82, 4.53	0.133
Sick/disabled/unable to work/others	0.82	0.47, 1.43	0.476	1.24	0.63, 2.42	0.530
**Residence**						
Rural informal	1	Reference	-	1	Reference	-
Rural farms	1.18	0.83, 1.67	0.360	1.03	0.70, 1.51	0.889
Urban	1.84	1.44, 2.34	< 0.001	1.70	1.29, 2.23	< 0.001
**Drug use (past 3 months)**						
No	1	Reference	-	1	Reference	-
Yes	3.11	2.54, 3.80	< 0.001	2.79	2.25, 3.46	< 0.001
**Psychological distress**						
No	1	Reference	-	1	Reference	-
Yes	1.46	1.20, 1.78	< 0.001	1.42	1.16, 1.75	< 0.001

OR, odds ratio; CI, confidence interval.

**TABLE 4:** Associations with hazardous, harmful or dependent alcohol consumption amongst women.

Variable	Simple logistic regression			Multiple logistic regression		
	Crude OR	95% CI	*p*	Adjusted OR	95% CI	*p*
**Age in years**						
15–24	1	Reference	-	1	Reference	-
25–34	1.72	1.24, 2.36	< 0.001	1.72	1.25, 2.36	< 0.001
35–44	1.00	0.69, 1.47	0.982	1.01	0.69, 1.47	0.962
45–54	0.93	0.64, 1.34	0.68	0.88	0.61, 1.27	0.502
55 or more	0.38	0.24, 0.59	< 0.001	0.4	0.25, 0.63	< 0.001
**Education**						
Grade 0–7	1	Reference	-	1	Reference	-
Grade 8–11	1.54	1.17, 2.02	0.002	1.13	0.83, 1.53	0.455
Grade 12 or more	1.15	0.89, 1.48	0.285	0.82	0.62, 1.09	0.174
**Population group**						
African black people	1	Reference	-	1	Reference	-
mixed race	2.32	1.77, 3.02	< 0.001	1.98	1.49, 2.63	< 0.001
Indian or Asian people	0.31	0.15, 0.61	< 0.001	0.27	0.14, 0.55	< 0.001
White people	0.9	0.56, 1.44	0.668	0.95	0.58, 1.56	0.854
**Employment status**						
Employed/self-employed	1	Reference	-	-	-	-
Unemployed	0.90	0.68, 1.19	0.460	-	-	-
Student/pupil/learner	0.71	0.43, 1.18	0.189	-	-	-
Sick/disabled/unable to work/others	0.54	0.18, 1.65	0.280	-	-	-
**Residence**						
Rural informal	1	Reference	-	1	Reference	-
Rural farms	3.52	2.13, 5.82	< 0.001	2.57	1.51, 4.38	< 0.001
Urban	3.28	2.35, 4.57	< 0.001	2.87	1.99, 4.14	< 0.001
**Drug use (past 3 months)**						
No	1	Reference	-	1	Reference	-
Yes	3.18	2.15, 4.69	< 0.001	2.91	1.94, 4.36	< 0.001
**Psychological distress**						
No	1	Reference	-	1	Reference	-
Yes	1.69	1.29, 2.20	< 0.001	1.68	1.27, 2.22	< 0.001

OR, odds ratio; CI, confidence interval.

### Associations with drug use

In adjusted logistic regression analysis, men having Grade 8–11 education, who are mixed race and unemployed and have HHDA use were positively and middle and older age (25 years and older) and a student or learner were negatively associated with past 3-month drug use. Women who are mixed race, Indians or Asians and have HHDA use were positively and older age (45 years and older) were negatively associated with past 3-month drug use (see Tables 5 and 6).

**TABLE 5:** Associations with drug use amongst men.

Variable	Simple logistic regression			Multiple logistic regression		
	Crude OR	95% CI	*p*	Adjusted OR	95% CI	*p*
**Age in years**						
15–24	1	Reference	-	1	Reference	-
25–34	1.19	0.95, 1.50	0.126	0.81	0.62, 1.04	0.102
35–44	0.75	0.59, 0.95	0.019	0.53	0.41, 0.70	< 0.001
45–54	0.58	0.43, 0.78	< 0.001	0.4	0.29, 0.56	< 0.001
55 or more	0.36	0.27, 0.48	< 0.001	0.26	0.19, 0.35	< 0.001
**Education**						
Grade 0–7	1	Reference	-	1	Reference	-
Grade 8–11	2.01	1.64, 2.47	< 0.001	1.52	1.20, 1.92	< 0.001
Grade 12 or more	1.16	0.95, 1.42	0.141	0.93	0.74, 1.19	0.581
**Population group**						
African black people	1	Reference	-	1	Reference	-
mixed race	1.31	1.03, 1.67	0.027	1.32	1.03, 1.69	0.029
Indian or Asian people	0.63	0.43, 0.93	0.019	0.79	0.52, 1.18	0.242
White people	0.59	0.41, 0.85	0.005	0.83	0.56, 1.24	0.362
**Employement status**						
Employed/self-employed	1	Reference	-	1	Reference	-
Unemployed	1.34	1.11, 1.62	0.002	1.27	1.02, 1.58	0.034
Student/pupil/learner	0.7	0.52, 0.93	0.015	0.52	0.35, 0.76	< 0.001
Sick/disabled/unable to work/others	0.77	0.43, 1.36	0.363	0.99	0.51, 1.90	0.965
**Residence**						
Rural informal	1	Reference	-	-	-	-
Rural farms	1.05	0.75, 1.46	0.778	-	-	-
Urban	1.24	0.98, 1.55	0.072	-	-	-
**Hazardous/harmful/dependent alcohol use**						
No	1	Reference	-	1	Reference	-
Yes	3.12	2.57, 3.78	< 0.001	2.81	2.29, 3.46	< 0.001
**Psychological distress**						
No	1	Reference	-	1	Reference	-
Yes	1.29	1.02, 1.62	0.031	1.22	0.96, 1.53	0.098

OR, odds ratio; CI, confidence interval.

**TABLE 6:** Associations with drug use amongst women.

Variable	Simple logistic regression			Multiple logistic regression		
	Crude OR	95% CI	*p*	Adjusted OR	95% CI	*p*
**Age in years**						
15–24	1	Reference	-	1	Reference	-
25–34	0.87	0.64, 1.18	0.367	0.85	0.63, 1.16	0.308
35–44	0.72	0.50, 1.03	0.069	0.71	0.50, 1.01	0.054
45–54	0.68	0.45, 1.02	0.061	0.64	0.43, 0.96	0.032
55 or more	0.6	0.43, 0.84	0.003	0.57	0.40, 0.80	< 0.001
**Education**						
Grade 0–7	1	Reference	-	-	-	-
Grade 8–11	0.94	0.68, 1.26	0.624	-	-	-
Grade 12 or more	1.09	0.83, 1.44	0.523	-	-	-
**Population group**						
African black people	1	Reference	-	1	Reference	-
mixed race	1.84	1.36, 2.62	< 0.001	1.70	1.25, 2.30	< 0.001
Indian or Asian people	1.25	0.61, 2.57	0.548	1.41	0.68, 2.91	0.355
White people	1.68	1.08, 1.62	0.022	1.94	1.22, 3.07	0.005
**Employment status**						
Employed/self-employed	1	Reference	-	-	-	-
Unemployed	0.99	0.74, 1.31	0.922	-	-	-
Student/pupil/learner	1.14	0.79, 1.63	0.482	-	-	-
Sick/disabled/unable to work/others	0.89	0.41, 1.95	0.775	-	-	-
**Residence**						
Rural informal	1	Reference	-	-	-	-
Rural farms	1.17	0.63, 2.19	0.615	-	-	-
Urban	1.2	0.85, 1.71	0.301	-	-	-
**Hazardous/harmful/dependent alcohol use**						
No	1	Reference	-	1	Reference	-
Yes	3.43	2.37, 4.95	< 0.001	3.08	2.11, 4.49	< 0.001
**Psychological distress**						
No	1	Reference	-	-	-	-
Yes	1.33	0.98, 1.81	0.071	-	-	-

OR, odds ratio; CI, confidence interval.

## Discussion

Compared to previous national population-based surveys in 2008 (9.0% HHDA^10^) and 2012 (4.4% past 3-month drug use^14^) and a national survey in Kenya (6.7% HHDA),^9^ this national survey in 2017 showed higher rates of HHDA (10.3%) and any past 3-month drug use (8.6%) (see Table 7). Similarly, Harker et al.^24^ found an increase of opioid use disorder treatment admissions from 16.1% in 2012 to 20.0% in 2017 in South Africa. Reasons for the overall slight increase of HHDA and increase in any drug use in South Africa from 2008 or 2012 to 2017 need further research.^25^

**TABLE 7A:** Prevalence of hazardous, harmful or dependent alcohol consumption and drug use across national surveys in South Africa.

Study year	Hazardous, harmful or dependent alcohol use			Any drug use		
	Total	Male	Female	Total	Male	Female
	
	%	%	%	%	%	%
2008^15^	9.0	17.0	2.9	3.3^26^	-	-
2012^14^	-	-	-	4.4	7.9	1.3
2017	10.3	16.5	4.6	8.6	13.3	4.1

**TABLE 7B:** Prevalence of hazardous, harmful or dependent alcohol consumption and drug use across national surveys in South Africa.

Study year	Drug use						
	Cannabis	Cocaine	Amphetamine	Inhalants	Sedatives	Hallucinogens	Opiates

	Total	Total	Total	Total	Total	Total	Total

	%	%	%	%	%	%	%
2008^26^	3.2	0.7	0.8	0.8	0.7	0.7	0.7
2012^14^	4.0	0.3	0.3	0.2	0.4	0.1	0.1
2017	7.8	1.8	1.5	1.3	1.7	1.2	1.2

In agreement with previous studies,^6,7,8,14,26,27^ this study found that male sex increased the odds and older age decreased the odds of HHDA and drug use. Sex-specific role expectations and norms, such as associating drinking alcohol and drug use with masculinity, may be related to the male preponderance of HHDA and drug use.^7,28^ In older age, in this study amongst women, a reduction of HHDA may be expected because the tolerance towards alcohol reduces with ageing.^29^

Amongst different population or ethnic groups in South Africa, mixed race women had significantly higher odds for HHDA and drug use. This result concurs with previous studies in South Africa for both mixed race women and men.^10,14^ It is possible that people of mixed race (coloured) are exposed to more stressors than other population groups contributing to higher rates of substance use. Whilst previous research showed an association between lower education and lower socioeconomic status,^10,30,31^ this study did not find that educational level was associated with HHDA and drug use amongst women, whilst drug use amongst men with higher education was positively associated with HHDA and drug use. The findings amongst women are interesting and warrant further investigation to fully understand the change. Amongst men with higher education, the positive association could be explained because of rapid modernisation, which strongly correlates with drug use.^32^ As South Africa progresses from apartheid, there are an increasing number of people entering the higher education and middle-upper income bracket.

On the other hand, amongst men, unemployment increased the odds of drug use in this study, which is in line with a previous study in South Africa.^26^ As stated by Peltzer et al:^32^

[*U*]se of drugs may be functional as it provides a form of release or escape not only for large numbers of unemployed (especially young men) who may also feel they are unemployable. (p. 2228)

Consistent with previous research findings,^16,17,33^ this study found strong associations between drug use, psychological distress and HHDA and drug use. This confirms the comorbity between HHDA and drug use and psychological distress, but comorbidity between drug use and HHDA but not psychological distress. Reasons for the comorbidity between HHDA and drug use may lie in the codependence risk of the substances used. Public health interventions should be directed at integrating drug use and psychological distress prevention in persons with HHDA.

### Study limitations

This study was limited by its cross-sectional design and self-report of data, including substance use. A further limitation was that in this household survey, populations using heavy substance, such as military personnel, homeless or institutionalised persons, were not included.^34^

## Conclusion

In this large national population-based survey amongst persons 15 and older in 2017 in South Africa, about one in 10 participants engaged in HHDA and drug use, with several sociodemographic (male sex, middle age, higher education, being unemployed, mixed race, urban residence) and health indicators (substance use and psychological distress), was identified to be associated with HHDA and/or any drug use.
